# Supplementary material for: Direct visualization of emergent metastatic features within an ex vivo model of the tumor microenvironment
Source: Life Sci Alliance. 2024 Oct 17;8(1):e202403053. doi: 10.26508/lsa.202403053 (PMC11487089; doi:10.26508/lsa.202403053)
Supplement: Supplementary file 5 [file LSA-2024-03053_TableS1.docx]

| <!--Col Count:6-->Condition | ***J_C_***_1,_***_C_***_1_ | ***J_C_***_1,_***_C_***_2_ | ***J_C_***_2,_***_C_***_2_ | ***J_C_***_1,_***_ECM_*** | ***J_C_***_2,_***_ECM_*** |
| --- | --- | --- | --- | --- | --- |
| **Increased Invasion** | 16 | 16 | 16 | 2 | 11 |
| **Decreased epithelial adhesion** | 24 | 16 | 8 | 11 | 11 |
| **Both** | 24 | 16 | 8 | 2 | 11 |

Table S1. **CPM parameters for different conditions.** *J_C_*_1,_*_C_*_1_ is the surface energy between to cells or a cell and the ECM. Higher surface energies lead to lower affinity. C1: core cells. C2: cortical cells. ECM: Substrate (extracellular matrix).
