# Supplementary material for: Direct visualization of emergent metastatic features within an ex vivo model of the tumor microenvironment
Source: Life Sci Alliance. 2024 Oct 17;8(1):e202403053. doi: 10.26508/lsa.202403053 (PMC11487089; doi:10.26508/lsa.202403053)
Supplement: Supplementary file 6 [file LSA-2024-03053_TableS2.docx]

| <!--Col Count:3-->Parameter | Value | Units |
| --- | --- | --- |
| **Temperature** | 37 | ºC |
| **Population size** | 100 | cells |
| **Target cell size** | 40 | pixels |
| **Dimensions** | 200x200 | pixels |
| **Boundary conditions** | Periodic | N/A |

Table S2. Additional CPM parameters.
